# K*i*DoQ: using docking based energy scores to develop ligand based model for predicting antibacterials

**DOI:** 10.1186/1471-2105-11-125

**Published:** 2010-03-11

**Authors:** Aarti Garg, Rupinder Tewari, Gajendra PS Raghava

**Affiliations:** 1 Bioinformatics Centre, Institute of Microbial Technology, Sector-39A, Chandigarh, India; 2 Department of Biotechnology, Panjab University, Chandigarh, India

## Abstract

**Background:**

Identification of novel drug targets and their inhibitors is a major challenge in the field of drug designing and development. Diaminopimelic acid (DAP) pathway is a unique lysine biosynthetic pathway present in bacteria, however absent in mammals. This pathway is vital for bacteria due to its critical role in cell wall biosynthesis. One of the essential enzymes of this pathway is dihydrodipicolinate synthase (DHDPS), considered to be crucial for the bacterial survival. In view of its importance, the development and prediction of potent inhibitors against DHDPS may be valuable to design effective drugs against bacteria, in general.

**Results:**

This paper describes a methodology for predicting novel/potent inhibitors against DHDPS. Here, quantitative structure activity relationship (QSAR) models were trained and tested on experimentally verified 23 enzyme's inhibitors having inhibitory value (*K*_i_) in the range of 0.005-22(mM). These inhibitors were docked at the active site of DHDPS (1YXD) using AutoDock software, which resulted in 11 energy-based descriptors. For QSAR modeling, Multiple Linear Regression (MLR) model was engendered using best four energy-based descriptors yielding correlation values *R*/*q*^2 ^of 0.82/0.67 and MAE of 2.43. Additionally, Support Vector Machine (SVM) based model was developed with three crucial descriptors selected using F-stepping remove-one approach, which enhanced the performance by attaining *R*/*q*^2 ^values of 0.93/0.80 and MAE of 1.89. To validate the performance of QSAR models, external cross-validation procedure was adopted which accomplished high training/testing correlation values (*q*^2^/*r*^2^) in the range of 0.78-0.83/0.93-0.95.

**Conclusions:**

Our results suggests that ligand-receptor binding interactions for DHDPS employing QSAR modeling seems to be a promising approach for prediction of antibacterial agents. To serve the experimentalist to develop novel/potent inhibitors, a webserver "K*i*DoQ" has been developed http://crdd.osdd.net/raghava/kidoq, which allows the prediction of *K*_i _value of a new ligand molecule against DHDPS.

## Background

An escalating magnitude of drug resistance among bacterial pathogens has been installing a serious threat on the public health and economy of the developed world. A survey report has suggested that the direct cost to US economy alone due to drug resistant bacterial infection is around $4-$5 billion annually [1-3]. Even for pharmaceuticals companies, it turns out to be a heart-dying situation that after investing ~$800 million and about 15 years of atrocious labor to introduce a drug in the market, the pathogens already attains resistance against the drug. Therefore, there is an urgent need to recognize new inhibitors against novel and/or known targets. Undoubtedly, well-established bacterial targets i.e. cell wall and membrane biosynthesis, protein biosynthesis, nucleic acid etc always the first choice for developing antibacterials. The recent trend in this direction indicates that researchers are looking for novel targets alongside to discover new classes of inhibitors/antibiotics.

**Figure 1:**
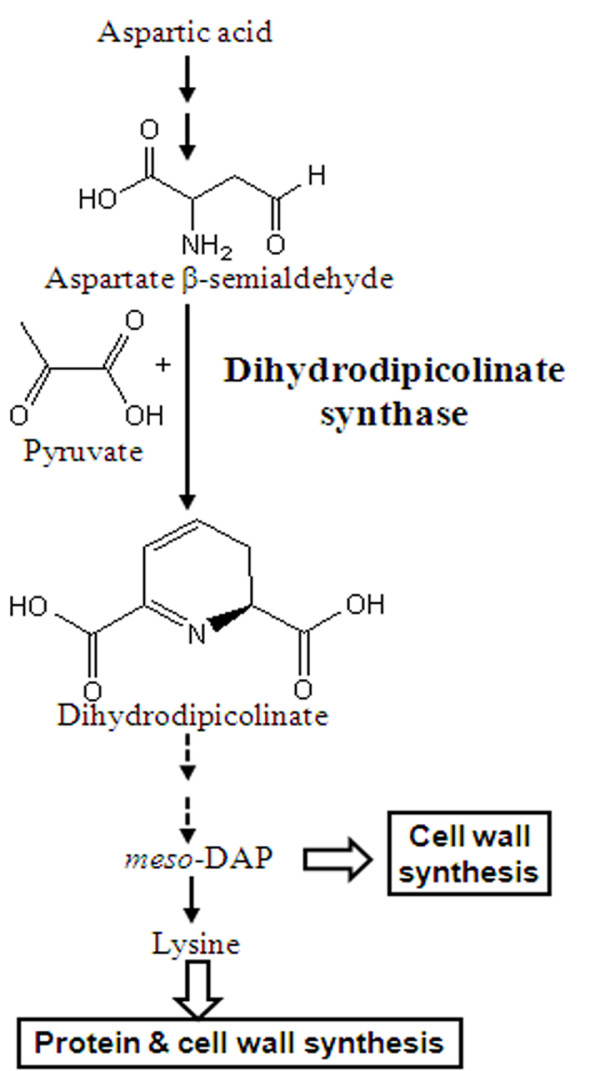
**Enzymatic action of DHDPS leads to the biosynthesis of bacterial cell wall and protein components**. Figure 1 shows the action of DHDPS enzyme involved in protein and cell wall synthesis process.

The amino acids biosynthetic pathways specifically lysine pathway has gained special attention because of its potential role in bacterial cell wall and protein synthesis [4,5]. The D, L-diaminopimelic acid (*meso*-DAP), an important intermediate in the biosynthetic pathway of lysine is crucial in cross-linking peptidoglycan chains to provide strength and rigidity to the bacterial cell wall (known as DAP pathway). The absence of this pathway in mammalian system suggests that specific inhibitors of this biosynthetic pathway may be a valuable for developing novel classes of antibacterial agents. In this study, we explored DHDPS enzyme of the pathway, which catalysis condensation of pyruvate and aspartate semialdehyde to form DHDP. Figure 1 shows the established DAP pathway for DAP and lysine biosynthesis. The enzyme is encoded by *dapA *gene, which has been cloned and expressed from several strains, including *Thermatoga maritima*, *Corynebacterium glutamicum, Mycobacterium tuberculosis *and *Bacillus anthracis*. The three-dimensional structures of DHDPS enzyme from *Escherichia coli*, *Staphylococcus aureus, M. tuberculosis *and *B. anthracis *enzymes with substrate pyruvate and without have been reported [6-18].

The antibacterial identification using experimental techniques is invariably very expensive, requires extensive pains and labor. Therefore, *in silico *techniques, which have the power to cut down these unavoidable steps, would be valuable. In recent years, *in silico *techniques like quantitative structure activity relationship (QSAR) and molecular docking are gaining high popularity in the drug discovery [19-21]. Both these methodologies allow the identification of probable lead candidates expeditiously prior to chemical synthesis and characterization, thereby, making the process more cost effective [22,23].

In the present study, we attempt to integrate power of two *in silico *potential techniques: QSAR and molecular docking by using docking generated energy-based descriptors for building QSAR models. Using this strategy, the information regarding binding mode of ligands in the active site is accumulated which would in turn assist the accurate prediction of better inhibitor with improved *K*_i _values. To facilitate this we also developed a web-interface to help experimentalist working in the field of designing novel inhibitors against DHDPS enzyme.

## Results

**Figure 2:**
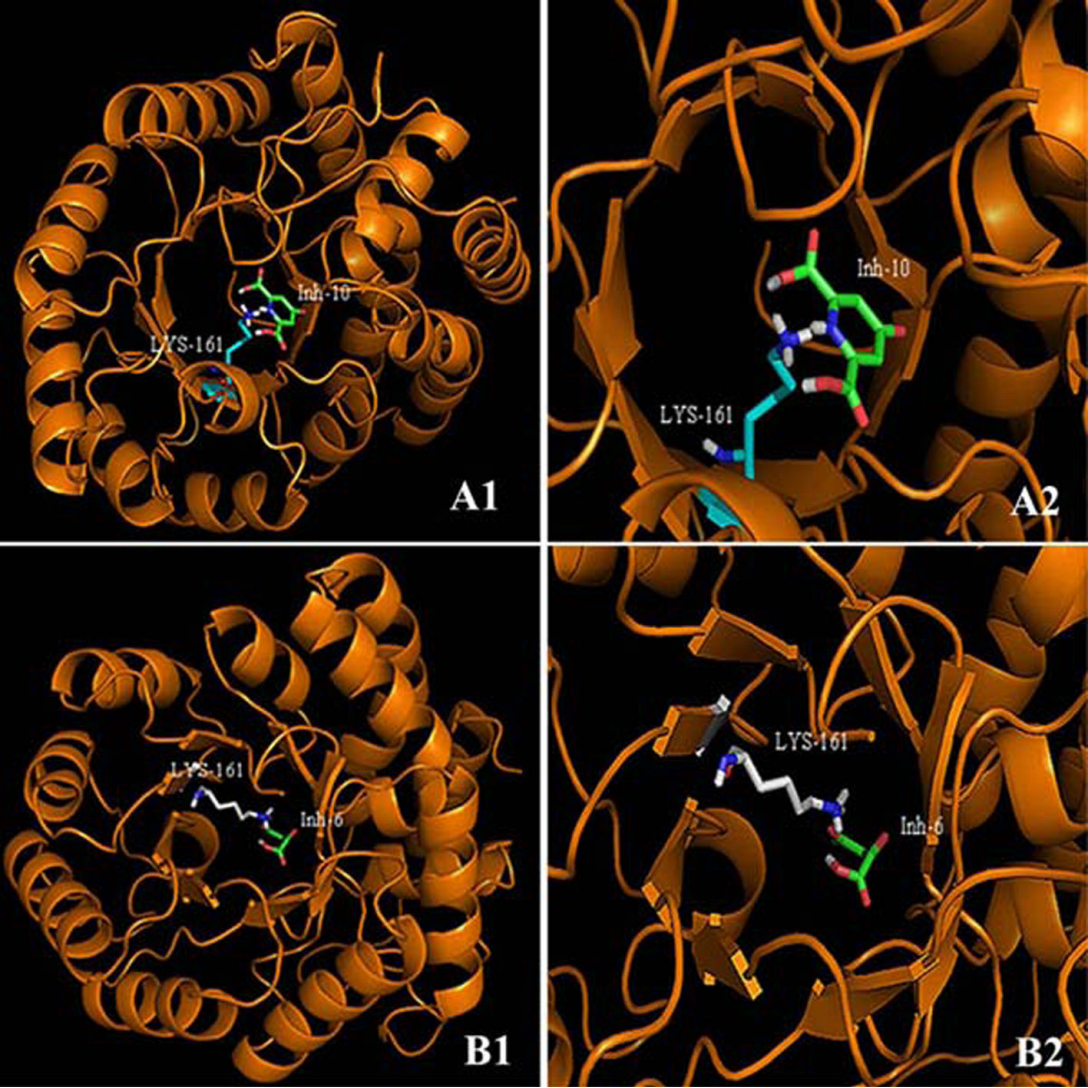
**View of docked Inh-10 (A1 and A2) and Inh-6 (B1 and B2) at the active binding site of DHDPS**. Figure 2 shows the docked conformation of inh-10 and inh-6 in active site of DHDPS where protein is shown in secondary structure and inh-10, inh-6 is represented in ball and stick model.

For the docking of 23 inhibitors, *E. coli *DHDPS crystal structure stored in the PDB file 1YXD was retrieved. The crystal structure of DHDPS consisted of two similar chains (A and B) with inhibitor bound at allosteric site [13]. The water molecules and inhibitor were removed using PYMOL software and chain A was considered for the docking purpose. The python scripts were used for carrying out automated flexible docking of 23 inhibitors on the predefined and experimentally characterized binding pocket, where the residue LYS161 being particularly very important. Hence, it's important to consider the flexibility of LYS161 and the inhibitors, while performing docking. Figure 2 shows the docking of two inhibitors: Inh-6 (having minimum *K*_i _value) and Inh-10 (with maximum *K*_i _value) at the active site of DHDPS enzyme. In order to validate our docking methodology another crystal structure of *E. coli *DHDPS (3DU0) with substrate bound at the active site was obtained from PDB. The enzyme 1YXD could not be used as it enclosed bound conformation of an allosteric inhibitor (*S*)-Lysine. Since crystals were remarkably similar (RMSD value of 0.15Å), therefore, the same procedure for the docking of pyruvate was adopted which resulted in very slight variation in the RMSD value of 0.31 Å. Hence, the docking protocol adopted in the present study was able to reproduce the conformation comparable to the crystal structure with substrate at active site. Additionally, analysis of 10 docked poses of substrate generated by AutoDock software was also carried out. In Additional File 1: table S1 we have shown the values of free binding energies and RMSDs in the inreasing order of ranking. It was observed that RMSD value for the fifth ranked pose was lesser in comparison with the pose with best and minimum free binding energy. We also calculated the pair-wise corelation between free binding energy and RMSD, resulted in *R *value of 0.81, which reveals that there exists correlation between free binding energy and RMSD values, however not the ideal or perfect one. Therefore, it's not always true that the pose with the lowest binding energy is the one with the lowest RMSD to the crystal structure. Ofcourse, one can validate or check the RMSD values for a single ligand system with bound crystal structure known. However, during virtual screening procedure with large number of unknown structures to dock, it's practically impossible to obtain the RMSD values. Therefore, in such cases, it has been shown in the past that the compounds with the lowest binding energies are generally considered as potential hits.

It's important to mention that in general, after docking, AutoDock computes 11 types of energy values i.e. - i) Estimated free energy of binding (E_FreeBind_); ii) Final Intermolecular Energy (E_InterMol_), which is the sum of 4 energies such as (iii) vdW + Hbond + desolv Energy (E_VHD_), (iv) Electrostatic Energy (E_Elec_), (v) Moving Ligand-Fixed Receptor (E_MLFR_), and (vi) Moving Ligand-Moving Receptor (E_MLMR_); vii) Final Total Internal Energy (E_FTot_), again the sum of 2 energy values such as (viii) Internal Energy Ligand (E_IntL_), and (ix) Internal Energy Receptor (E_IntR_); (x) Torsional Free Energy (E_Tors_) and (xi) Unbound System's Energy (E_Unb_). Finally, 11 types of energy values based descriptors were then used as independent variables for QSAR modeling.

To obtain significant and non-correlated variables from the above-mentioned 11 descriptors, a statistical package, STATISTICA, was used. Indeed all the descriptors were highly significant, showing the *p *< 0.05. To filter out correlated descriptors, pair-wise correlation coefficient at the cut-off value of 0.9 was imposed. The two variables namely E_Unb _and E_IntL _yielded the pair-wise correlation values > 0.9 with E_FTot _and therefore filtered out from the further analysis (Table 1).

**Table 1:** Matrix showing the pair-wise correlation values for docking generated 11 energy-based descriptors

	*E*_*FreeBind*_	*E*_*InterMol*_	*E*_*VHD*_	*E*_*Elec*_	*E*_*MLFR*_	*E*_*MLMR*_	*E*_*FTot*_	*E*_*IntL*_	*E*_*IntR*_	*E*_*Tors*_	*E*_Unb_
***E*_*FreeBind*_**	1.000	**0.846**	**0.800**	0.167	**0.776**	0.171	-0.181	-0.021	-0.450	-0.140	0.043
***E*_*InterMol*_**	**0.846**	1.000	**0.884**	0.183	**0.857**	0.312	-0.369	-0.155	-0.624	-0.458	-0.111
***E*_*VHD*_**	**0.800**	**0.884**	1.000	-0.031	**0.840**	0.124	-0.278	-0.141	-0.404	-0.374	-0.037
***E*_*Elec*_**	0.167	0.183	-0.031	1.000	**0.516**	-0.603	0.022	0.183	-0.425	-0.143	0.069
***E*_*MLFR*_**	**0.776**	**0.857**	**0.840**	**0.516**	1.000	-0.222	-0.225	-0.020	-0.577	-0.397	0.007
***E*_*MLMR*_**	0.171	0.312	0.124	-0.603	-0.222	1.000	-0.279	-0.251	-0.120	-0.136	-0.219
***E*_*FTot*_**	-0.181	-0.369	-0.278	0.022	-0.225	-0.279	1.000	**0.935**	0.324	-0.380	**0.938**
***E*_*IntL*_**	-0.021	-0.155	-0.141	0.183	-0.020	-0.251	**0.935**	1.000	-0.033	-0.498	**0.956**
***E*_*IntR*_**	-0.450	-0.624	-0.404	-0.425	-0.577	-0.120	0.324	-0.033	1.000	0.256	0.093
***E*_*Tors*_**	-0.140	-0.458	-0.374	-0.143	-0.397	-0.136	-0.380	-0.498	0.256	1.000	-0.480
***E*_*Unb*_**	0.043	-0.111	-0.037	0.069	0.007	-0.219	**0.938**	**0.956**	0.093	-0.480	1.000

**Figure 3:**
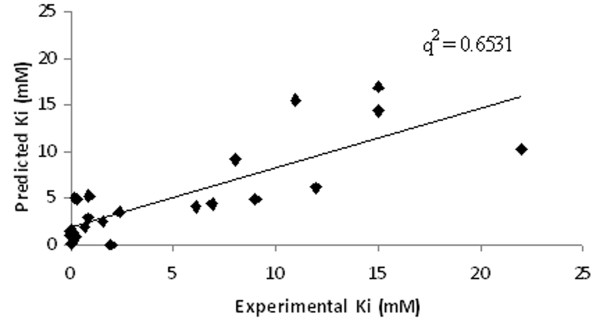
**Comparison between actual and predicted Ki values for MLR model generated using descriptors selected at pair-wise correlation cut-off value 0.9**. Figure 3 depict the experimental and predicted Ki value in X and Y direction respectively with q^2 ^value 0.6531 using MLR model.

**Figure 4:**
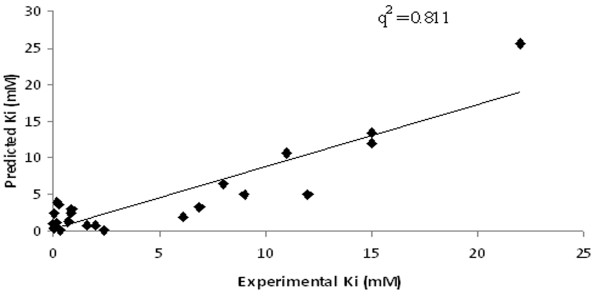
**The correlation between actual and predicted Ki values for SVM model generated using variables selected at pair-wise correlation cut-off value 0.9**. Figure 4 illustrate the experimental and predicted Ki value in X and Y direction respectively with q^2 ^value 0.811 using SVM model.

Using MLR, a QSAR based model was generated using 4 variables namely, E_FreeBind_, E_Elec_, E_IntR_, and E_Tors _which accomplished correlation (*R*/*q*^2^) values of 0.81/0.65 with MAE of 2.61 (Table 2). Though model was able to obtain good correlation values however, *q*^2 ^value was observed to be very low (Figure 3). Next, SVM along with F-stepping variable selection approach was employed. During first cycle of F-stepping remove-one, an elimination of fifth descriptor i.e. E_MLFR _from the set of *n *= 9 and the development of SVM model using 8 remaining variables attained the best correlation *R*/*q*^2 ^values of 0.87/0.75 and MAE of 2.24 (C = 50, γ = 45) listed in Table 2. For the second cycle, the removal of 9^th ^descriptor i.e. E_IntR _further improved the correlation value to 0.91/0.81 showing reduction in MAE to 2.01 (C = 50, γ = 50). The next cycle however did not enhance the correlation values significantly as exclusion of E_MLMR _offered correlation values 0.90/0.81 and MAE of 2.16 (C = 75, γ = 50) therefore, making its absence or presence to elicit no influence on correlation values. The correlation between predicted and actual activity values is shown in Figure 4. In the subsequent cycles of variables selection, no improvement in correlation values was observed. Therefore, it can be deduced that 6 descriptors i.e. E_FreeBind_, E_InterMol_, E_VHD_, E_Elec_, E_FTot _and E_Tors _are important to predict the inhibitory activity values for the present dataset of 23 inhibitors against DHDPS. Further, an external cross-validation was carried out by randomly dividing 23 inhibitors dataset into three different sizes of training and test sets such as 21 and 2; 19 and 4; 17 and 6 respectively. The highest correlation *q*^2^/*r*^2 ^values of 0.81/0.97 (an average of 8-9 best models) was obtained for the largest training and smallest test sets of 21 and 2 inhibitors (Table 3). An increase in the size of test set with corresponding decrease in training set size reduced the *r*^2 ^values along with slight reduction in *q*^2 ^values. The notion behind this splitting was to appraise a high predictive correlation values on the test set even when the size of training set was very low.

**Table 2:** Correlation values for MLR and SVM based QSAR models developed using descriptors selected at pair-wise correlation cut-off value 0.9

*Number of input variables*	*R*	***q***^2^	*MAE*
***Using MLR (23 inhibitors)***			

4	0.81	0.65	2.61

***Using SVM (23 inhibitors)***			

8	0.87	0.75	2.24
7	0.91	0.81	2.01
**6**	**0.90**	**0.81**	**2.16**
5	0.90	0.79	2.19

***Using SVM (20 inhibitors)***			

5	0.95	0.89	1.28

**Table 3:** Detailed results obtained during external cross-validation procedure using six descriptors with pair-wise correlation cut-off value below 0.9

Size of training set	*q*^2^	MAE	Size of test set	*r*^2^	MAE
21	0.81 ± 0.01	2.26 ± 0.09	2	0.97 ± 0.02	0.74 ± 0.23
19	0.76 ± 0.03	2.58 ± 0.12	4	0.94 ± 0.02	0.94 ± 0.15
17	0.73 ± 0.03	2.7 ± 0.08	6	0.80 ± 0.11	1.60 ± 0.36

**Figure 5:**
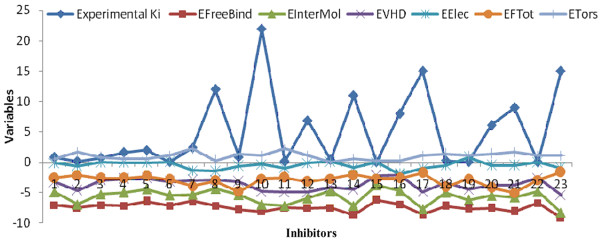
**The variations in the values of 6 energy-based input variables with respect to experimental Ki values**. Figure 5 shows the variation in energy descriptors with respect to Ki values.

**Figure 6:**
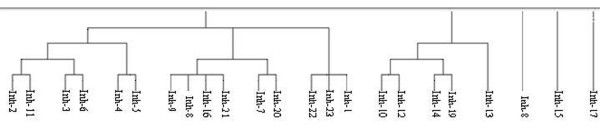
**The clustering of 23 inhibitors dataset**. Figure 6 illustrate the clustering graph of all 23 inhibitors where inh-8, inh-15 and inh-17 exist as singleton.

**Figure 7:**
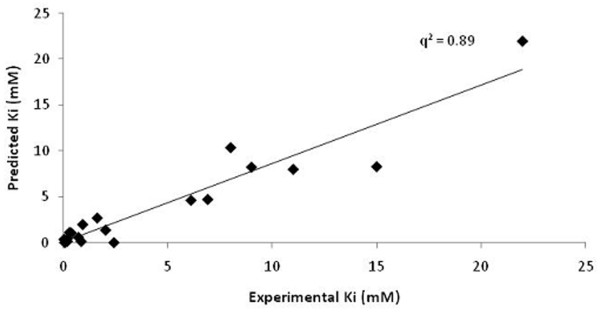
**Comparison between actual and predicted Ki values for SVM model developed using 20 inhibitors dataset**. Figure 7 depict the experimental and predicted Ki value in X and Y direction respectively with q^2 ^value 0.89.

Besides, pair-wise correlation coefficient values listed in Table 4 between *K*_i _and energy-based descriptors for 23 inhibitors were calculated. Surprisingly, three descriptors such as E_FreeBind_, E_InterMol_, and E_VHD _from the finally selected 6 energy-based descriptors (described earlier) showed high fluctuations with respect to *K*_i _values (Figure 5), which in turn have higher pair-wise correlation coefficient values (irrespective of signs). On the other hand, the variables i.e. E_FTot _and E_Tors _observed to be neutral, indeed provided low correlation values of 0.20 and 0.075 respectively. The E_MLFR_, which showed high correlation value of 0.62 with *K*_i_, was removed in the first cycle of variables selection. These deviations prompted us to carry out the clustering of the dataset of 23 inhibitors using JChem software http://www.chemaxon.com/. As shown in Figure 6, all 23 inhibitors clustered into two unique groups however, three compounds Inh-8, Inh-15, Inh-17 were variable. Hence, to filter the noise that might be caused due to these 3 inhibitors, a QSAR model was again generated removing these 3 structures. Using F-stepping variable selection approach, 5 energy-based input variables i.e. E_FreeBind_, E_InterMol_, E_VHD_, E_IntL _and E_MLFR _generated a QSAR model attaining correlation *R*/*q*^2 ^values of 0.95/0.89 and MAE of 1.28 (Figure 7 and Table 2).

**Table 4:** Pair-wise correlation values for 11 energy-based descriptors with respect to Ki values

	*E*_*FreeBind*_	*E*_*InterMol*_	*E*_*VHD*_	*E*_*Elec*_	*E*_*MLFR*_	*E*_*MLMR*_	*E*_*FTot*_	*E*_*IntL*_	*E*_*IntR*_	*E*_*Tors*_	*E*_*Unb*_
*R*	(-)0.66	(-)0.53	(-)0.45	(-)0.44	(-)0.63	0.15	0.20	(-)0.01	0.59	(-)0.075	0.001

**Figure 8:**
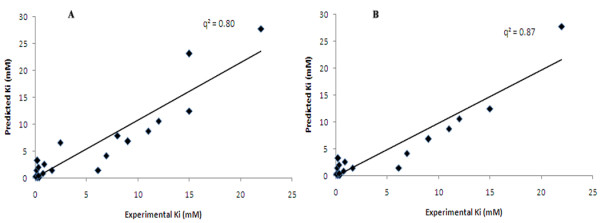
**Scatter plot between experimental versus predicted Ki values provided by highly non-correlated 3 energy values based SVM model for 23 inhibitors (A) and 20 inhibitors dataset (B)**. Figure 8 depict the experimental and predicted Ki value in X and Y direction respectively with q^2 ^value 0.80 and 0.87 for 23 and 20 inhibitors dataset respectively.

Additionally, QSAR modeling was also carried out using six non-correlated descriptors i.e. E_FreeBind_, E_Elec_, E_Ftot_, E_IntR_, E_MLMR _and E_Tors _having pair-wise correlation value less than 0.5. Using MLR, the QSAR model was developed with four types of input variables- E_FreeBind_, E_MLMR_, E_IntR_, and E_Tors_, which accomplished correlation (*R*/*q*^2^) values of 0.82/0.67 and MAE of 2.43 (Table 5). Hence, a small increase in correlation values in comparison to earlier MLR model (0.81/0.65 with MAE of 2.61), was observed. Further, SVM model was trained using six descriptors but the model attained poor correlation (*R*/*q*^2^) values of 0.67/0.40 and MAE of 2.91 (C = 200, γ = 1) signifying the presence of some descriptors ideally may not required for robust model generation. Thus, employing the F-stepping variable selection approach, the removal E_MLMR _from the set of *n *= 6 energy-based descriptors and using the 5 remaining descriptors for the SVM model development achieved best correlation *R*/*q*^2 ^values of 0.83/0.67 and MAE of 2.63 (C = 20, γ = 55) (Table 5). Next, filtering of E_Tors _enhanced the correlation (*R*/*q*^2^) value of model to 0.84/0.69 with reduction in MAE to 2.51 (C = 75, γ = 125). Then, QSAR modeling was carried out on the remaining four descriptors and the removal of E_FTot _augmented the correlation value to 0.93/0.80 with attenuation of MAE value to 1.89, a noteworthy enhancement. The correlation between predicted activities for the inhibitors and their actual experimental values is depicted in Figure 8A. There exists a good agreement between predicted and experimental activity values hence suggesting the robustness of QSAR model. Therefore, the three energy-based descriptors such E_FreeBind_, E_Elec_, and E_IntR _were imperative to predict the inhibitory activity values. Interestingly, the performance of three descriptor based QSAR model was found to be better in comparison to the six descriptors based SVM model (0.90/0.81 and MAE of 2.16) which was described earlier using cut-off value of 0.9. Additionally, the removal of three outliers like Inh-7, Inh-16 and Inh-23, further enhanced the prediction efficiency of QSAR model by increasing the correlation (*R*/*q*^2^) values to 0.94/0.87 and reduction in MAE to 1.45 (Figure 8B). Further, high external training/testing cross-validated correlation values (*q*^2^/*r*^2^) in the range of 0.78-0.83/0.93-0.95 was attained by randomly splitting the dataset into several training sets for model building and independent testing on corresponding test sets (Table 6).

**Table 5:** Correlation values for MLR and SVM based QSAR models developed using non-correlated variables selected at the cut-off value 0.5.

*Number of input variables*	*R*	*q*^2^	*MAE*
***Using MLR (23 inhibitors)***			

4	0.82	0.67	2.43

***Using SVM (23 inhibitors)***			

5	0.83	0.67	2.63

4	0.84	0.69	2.51

**3**	**0.93**	**0.80**	**1.89**

***Using SVM (20 inhibitors)***			

3	0.94	0.87	1.45

**Table 6:** Results obtained during external cross-validation procedure using three non-correlated descriptors

Size of training set	*q*^2^	MAE	Size of test set	*r*^2^	MAE
21	0.83 ± 0.02	1.90 ± 0.07	2	0.95 ± 0.03	0.98 ± 0.09
19	0.81 ± 0.01	2.01 ± 0.08	4	0.94 ± 0.02	0.98 ± 0.37
17	0.78 ± 0.03	2.28 ± 0.24	6	0.93 ± 0.03	1.09 ± 0.24

In order to assess robustness and validation of the finally developed three descriptors based QSAR model, a bootstrap analysis for 100 runs by statistical sampling of the original dataset was also performed which yielded a higher *q*^2^_bootstap _value of 0.88 ± 0.029. Thus, higher and lower values of *q*^2^_bootstap _and standard deviation parameters comprehensively support the statistical validity of the presently developed QSAR models. Further, Y-randomization test was also carried out using shuffled activity dataset which resulted in poor performance i.e. nearly all of the *q*^2 ^values were < zero (*q*^2 ^ranged from -0.15 to -0.41 and MAE from 4.15 to 5.13), thereby signifying the consistency of QSAR model.

### 2D descriptors based QSAR modeling

To compare the performance of three energy-based QSAR model with simple 2D QSAR models, the 2D QSAR modeling with 14 non-correlated descriptors was also performed. The study was commenced by MLR modeling, which tried to establish structure-activity relationship using five descriptors i.e. MSD, PJI2, Jhetm, ALOGP2, and Me by attaining correlation *R*/*q*^2 ^values of 0.78/0.61 and MAE of 3.08 (Table 7). The performance of 2D descriptors based model was found to be lower in comparison with four energy-based MLR model described earlier (*R*/*q*^2 ^values of 0.82/0.67 and MAE of 2.43). Further, using all 14 non-correlated descriptors for the training of SVM model, a very poor correlation (*R) *value of 0.23 was observed. The removal of 7 descriptors: nBm, BLI, Jhetm, GATS1v, nHAcc, ALOGP and MATS3 m (after employing 7 cycles of F-stepping remove-one) and the training of SVM model with remaining 7 descriptors attained *R*/*q*^2 ^values of 0.77/0.57 and MAE of 3.26 (C = 25, γ = 25). During the next cycle, an exclusion of JGI2 (topological charge index) optimized the SVM model (C = 300, γ = 25) by achieving correlation *R*/*q*^2 ^values of 0.79/0.60 and MAE of 3.10. In the next cycle, the removal of nH (constitutional) descriptor improved the correlation value to 0.82/0.64 with reduction in MAE value to 2.68 (C = 200, γ = 25). Finally, elimination of Me constitutional descriptor and the development of QSAR model on the remaining 4 descriptors, which included two topological descriptors-MSD, PJI2, molecular property-ALOGP2 and Burden eigenvalues descriptor-BEHm1, yielded correlation *R*/*q*^2 ^values of 0.84/0.67 and MAE of 2.61 (C = 300, γ = 25). Hence, the performance of SVM based 2D QSAR model was found to be very low in comparison with three energy values based QSAR model developed using SVM.

**Table 7:** Performance of 2D QSAR based MLR and SVM models

*Number of input variables*	*R*	***q***^2^	*MAE*
***Using MLR***			

5	0.78	0.61	3.08

***Using SVM***			

7	0.77	0.57	3.26
6	0.79	0.60	3.10
5	0.82	0.64	2.68
4	0.84	0.67	2.61

### Implementation of webserver

**Figure 9:**
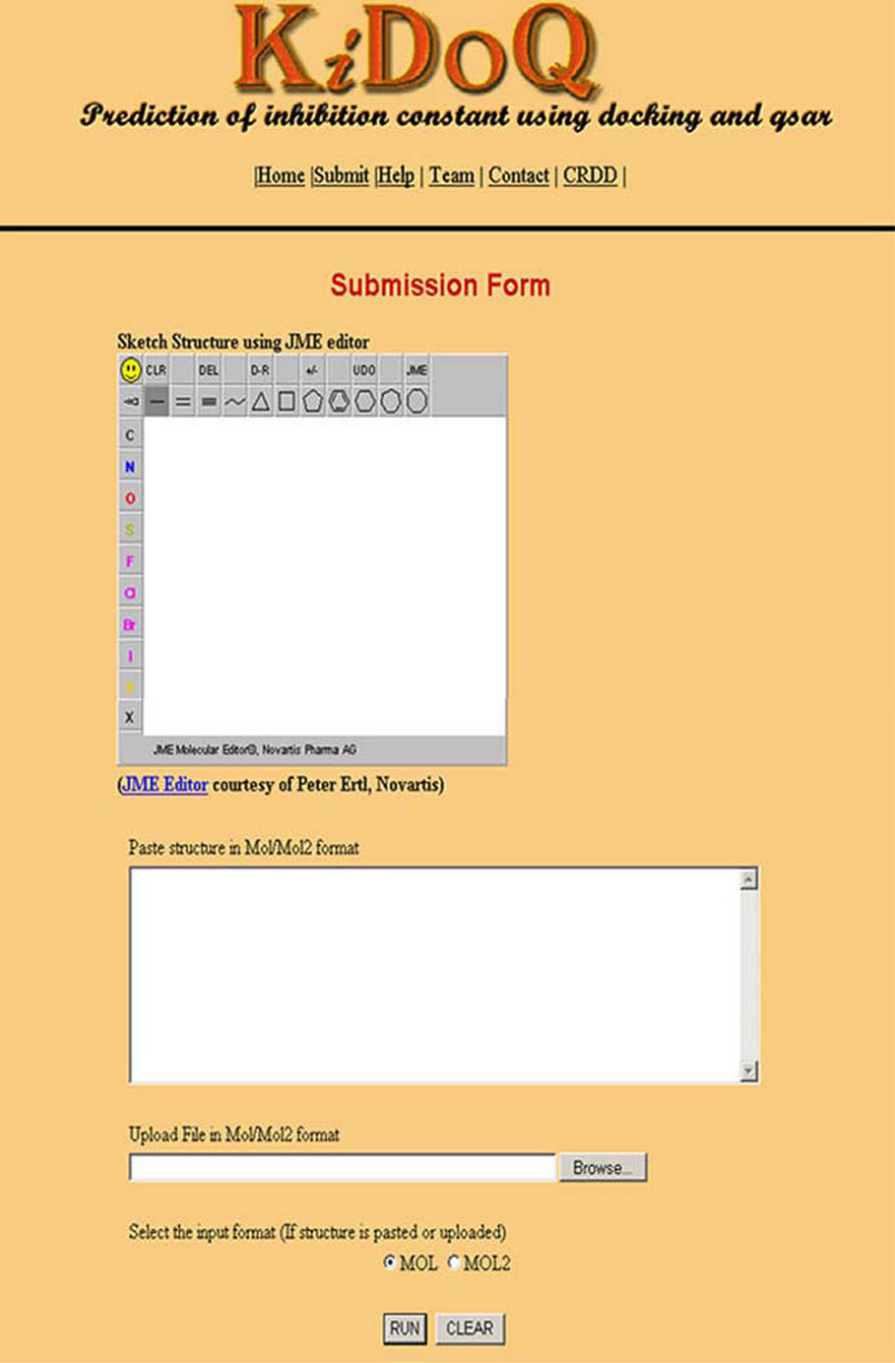
**A snapshot of submission page of KiDoQ webserver**. Figure 9 shows the screen shot of KiDoQ webserver.

**Figure 10:**
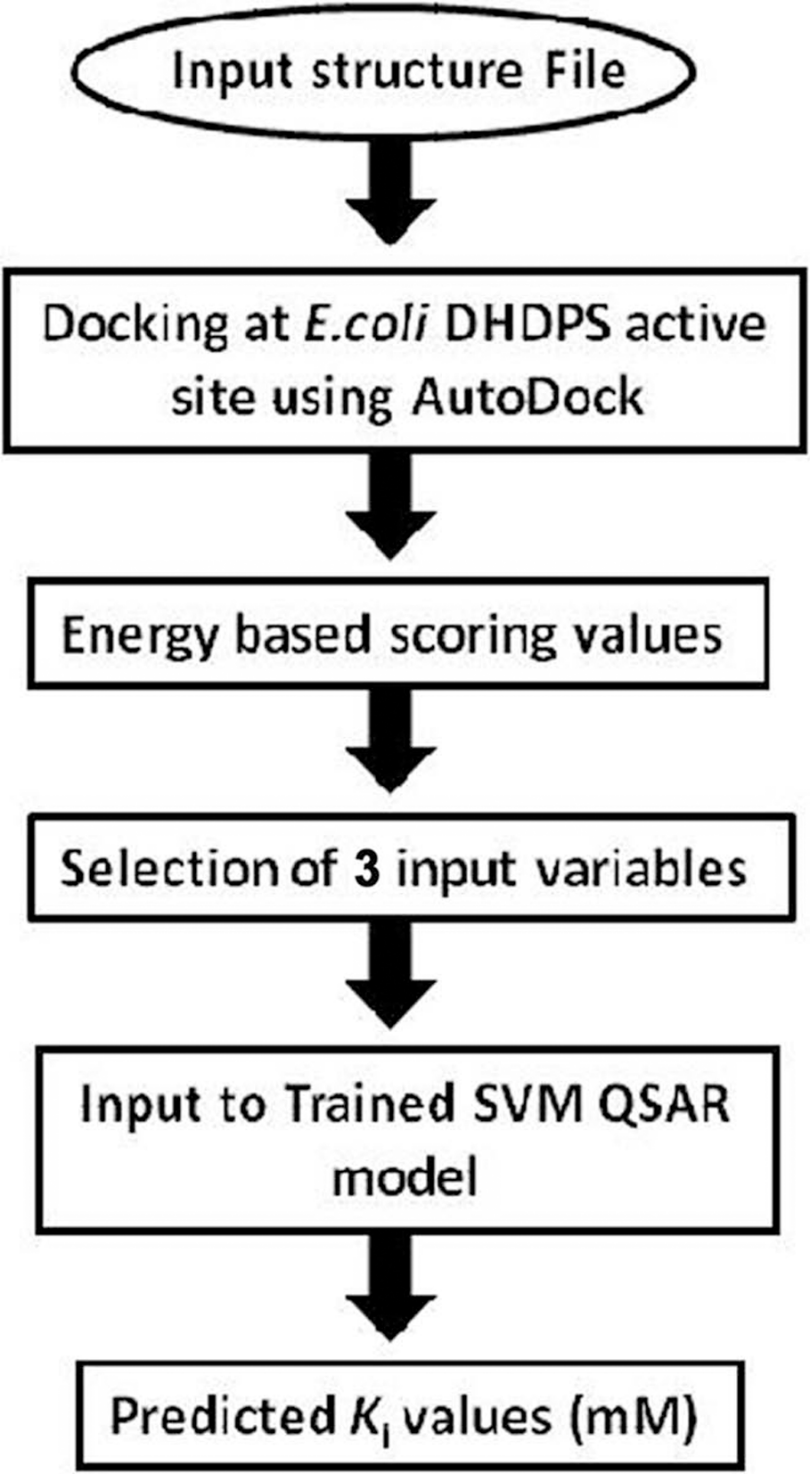
**Schematic flow for the working of KiDoQ webserver**. Figure 10 illustrate the workflow diagram of KiDoQ webserver.

We attempted to develop efficient QSAR model and based on these models, a web server "K*i*DoQ" (available at http://crdd.osdd.net/raghava/kidoq) using CGI-PERL and python scripts was developed. User can draw the structure of ligand molecule using JME editor incorporated on the server. The server also accepts input as mol/mol2 structure files pasted or uploaded on the server (Figure 9). The working flow of K*i*DoQ server is shown in Figure 10.

## Discussion

The QSAR modeling has been accepted as a promising methodology for lead identification. Nevertheless, if high-resolution target structure is available, then receptor structure based approach is often a first choice. The recent studies have shown the better performance of QSAR models even in the presence of target structure. However, simple QSAR approach can sometimes lead to false prediction if the collected data does not cover the complete property space or the selected 2D/3D descriptors are not reliable. Therefore, both techniques have their own advantages and limitations [19-23]. Keeping in view, the importance of docking and better performance of QSAR, we integrated both approaches by using docking generated energy-based scores as descriptors for QSAR modeling. The major benefit presumed by this integration would be an additional validation of the docking predicted inhibitors as bioactive or inactive by prediction of their bioactivity values using QSAR models henceforth, would facilitate in reduction of false positives.

In the present study, docking of the 23 experimentally known inhibitors of DHDPS at its active binding site resulted in 11 energy-based descriptors. For valid statistical results, it was imperative to restrict the maximal number of descriptors or to remove highly correlated ones, as presence of redundancy reduced the discriminating power of input variables, thereby reducing their worth in model development. Ideally, a regression model with *n *training set compounds and *k *descriptors may be acceptable only if *n *> 4*k *and for any of the *k *descriptors- i) the significance level *p *is < 0.05; ii) the pair-wise correlation coefficient should be < 0.9 [24,25]. Therefore, we also looked for statistically significant and non-correlated energy-based descriptors. All 11 types of energy-based descriptors were statistically significant but only 2 and 5 variables showed pair-wise correlation value > 0.9 and 0.5 respectively, resulting their removal necessary for rigorous QSAR modeling. As QSAR modeling for DHDPS enzyme's inhibitors is being carried out for the first time, therefore both linear (MLR) and non-linear (SVM) techniques were employed. In our study, the performance of SVM model was found to be much better in comparison with MLR model. The simple linear model was unable to handle the diversity of the present dataset; therefore, the cases where simple linear techniques fail, non-linear techniques could provide a better option.

Further, it was also noticed that the structural diversity of 23 inhibitors and redundancy among finally selected six descriptors resulted in wrong selection of input variables. Therefore, we removed highly diverse three structures and used remaining 20 inhibitors for QSAR modeling. Interestingly, the performance of model was found to be enhanced in comparison to the model developed using 23 inhibitors. Further, QSAR modeling carried out with highly non-correlated 3 descriptors i.e E_FreeBind_, E_Elec_, and E_IntR _(selected at the cut-off pair-wise correlation value of 0.5) provided better correlation values in comparison to the earlier six descriptors (selected at the cut-off pair-wise correlation value of 0.9) based QSAR model. Therefore, removal of redundant descriptors reduced the noise and enabled the better training of QSAR models. The three non-correlated descriptors appeared to be governing factors in establishing structure actvity relationship for DHDPS enzyme. One of the possible reasons for their selection was a higher pair-wise correlation value with respect to *K*_i _in comparison with other descriptors i.e. E_Ftot_, E_MLMR _and E_Tors _removed during QSAR modeling.

Among three descriptors, the value of E_FreeBind _based descriptor was found to be dependent on other two descriptors i.e. E_Elec_, and E_IntR _as well on other correlated energy based descriptors (such as E_VHD_). Generally, in the absence of receptor's flexibility E_IntR _remains constant and does not make any significant contribution to E_FreeBind_, however, the flexibility incorporates transformations leading to internal energy changes. In the present study, the changes in the value of E_IntR _were observed as the LYS161 was kept flexible. It was noticed that inhibitors with lower *K*_i _value were characterized by high negative E_IntR _values. These inhibitors included aliphatic compounds generally the pyruvate and aspartate semialdehyde analogues. On the other hand, inhibitors such as Inh-17, Inh-14, Inh-23 and Inh-10 with higher *K*_i _values exhibited lower negative E_IntR_. In view of this, we suggest E_IntR _based descriptor is an important discriminating variable for developing robust QSAR models. Further, E_Elec _was also found to be imperative as variations in the E_Elec _values was highly dependent on the number and type of receptor residues involved in establishing charge interactions with inhibitors. We observed that inhibitors such as Inh-9, Inh-11, Inh-18 and Inh-20 with strong electrostatic interactions with receptor exhibited strong binding, resulting in higher negative E_FreeBind _and E_Elec _values that in turn provided lower *K*_i _values. A few inhibitors such as Inh-17 and Inh-14 characterized by aromaticity, the strong electrostatic or *π-cationic *interactions though provided higher negative E_Elec _values however, at the cost of reasonable reduction in the E_IntR _values, which in turn provided higher *K*_i _values, in comparison to the aliphatic inhibitors and the ones with weak electrostatic interactions. In addition, it was also figured out that inhibitors i.e. Inh-1, Inh-3, Inh-4, Inh-5, Inh-6, Inh-12, Inh-13, Inh-15 and Inh-19 exhibited strong affinity to receptor albeit no or very weak electrostatic interactions were observed. This suggest that binding of inhibitors to DHDPS is not specifically dependent on electrostatic interactions, however other bonded and non-bonded interactions appeared to be playing important role, which in turn provided higher negative E_FreeBind _values and lower *K*_i _values (For details see Additional File 1).

As we have employed a complex procedure of using docking generated energy-based descriptors for QSAR modeling; therefore, it became imperative to compare the model performance with simple conventional 2D QSAR models. The SVM based 2D QSAR model achieved a poor correlation value of 0.84/0.67 in comparison with docking energy-based SVM model (0.93/0.80) indicating inadequacy of 2D descriptors in providing acceptable and robust QSAR model for dataset of 23 inhibitors. This low performance of 2D QSAR models may be due to presence of high structural diversity among the inhibitors that was not easily captured using simple 2D descriptors.

To conclude, the present strategy of predicting *K*_i _values using docking generated energy-based descriptors for QSAR modeling is a promising approach to predict potent inhibitors against DHDPS enzyme.

## Conclusions

In this study, we describe a new approach for prediction of antibacterial compounds that both take QSAR and docking strategy into its consideration. By using this approach, we get promising results instead of using these two strategies individually and develop a webserver called KiDoQ. This webserver will be helpful for better prediction of antibacterial compounds against dihydrodipicolinate synthase (DHDPS).

## Methods

### Inhibitors Dataset

The information regarding the experimentally known 23 inhibitors, classified as potent, moderate and slightly weak, was obtained from the literature [6-18]. The IUPAC names of these inhibitors along with *K*_i _values are shown in Table 8. Chem3D Ultra (v11.0), windows-based software was used for sketching the 2D structures for all inhibitors followed by cleaning and refinement in order to correct the accidentally distorted or unrealistic bond angles and lengths. The 2D structures were converted into 3D structures using CORINA software. Then each structure was energy minimized to give energetically preferred 3D structures.

**Table 8:** Dataset of 23 inhibitors along with their experimentally known *K*_i _values

S. No	Inhibitor IUPAC Name	*K*_i _values (mM)
Inh-1	2-oxobutanoate	0.83
Inh-2	2-oxoheptanedioate	0.17
Inh-3	2-oxopentanoate	0.7
Inh-4	3-bromo-2-oxopropanoate	1.6
Inh-5	3-fluoro-2-oxopropanoate	0.22
Inh-6	2,4-dioxopentanoic acid	0.005
Inh-7	(2R)-2-amino-3-(2-aminoethylsulfanyl)propanoic acid	2.4
Inh-8	(2S)-2-aminocyclopentan-1-one	12
Inh-9	(2R)-2-azaniumyl-4-hydroxy-4-oxobutanoate	0.09
Inh-10	4-oxo-1H-pyridine-2,6-dicarboxylic acid	22
Inh-11	2,6-dioxoheptanedioic acid	0.156
Inh-12	Dimethyl 4-oxo-1H-pyridine-2,6-dicarboxylate	6.9
Inh-13	Pyridine-2,6-dicarbonitrile	0.35
Inh-14	Pyridine-2,6-dicarboxylate	11
Inh-15	Oxaldehydate	0.028
Inh-16	(3R)-3-aminooxolan-2-one	8
Inh-17	Benzene-1,3-dicarboxylic acid	15
Inh-18	(2S)-2-amino-4-oxobutanoic acid	0.27
Inh-19	1-oxidopyridin-1-ium-2,6-dicarboxylic acid	0.06
Inh-20	(2S)-2-azaniumyl-3 sulfinopropanoate	6.1
Inh-21	(2S)-2-azaniumyl-5-hydroxy-5-oxopentanoate	9
Inh-22	4-oxobutanoic acid	0.3
Inh-23	(1R)-cyclohex-3-ene-1,3-dicarboxylic acid	15

### Docking energy-based descriptors

were calculated using automated docking software AutoDock (v.4.0) (AD) [26]. It is a suite of three C programs: i) AutoTors, which facilitates input of ligand coordinates; ii) AutoGrid, which precalculates a three-dimensional grid based on macromolecular coordinates; and iii) AutoDock, which performs a actual docking simulations. Before docking process, several separate pre-docking steps: ligand preparation, receptor preparation and grid map calculations were performed. The ligand and receptor preparation stage involved the addition of hydrogen atoms, computing charges, merging non-polar hydrogen atoms and defining AD4 atom types to ensure that atoms conformed to the AutoDock atom types. The information about rotatable torsion bonds that defines the bond flexibility was acquired. The ligands and receptor molecule preparation was followed by grid construction using AutoGrid module. During grid construction, atom types of the ligand, which acted as probes in the calculation of grid maps, were identified. The grid with default volume of 40 × 40 × 40 Å with a spacing of 0.375Å centered on the receptor was prepared. For conformational searches, the docking calculations using the genetic algorithm (GA) procedure with default parameters was performed.

### 2D QSAR modeling

DRAGON software was used for the calculation of 2D descriptors. For our dataset, the software calculated ~848 types of 2D descriptors categorized into different descriptor blocks such as constitutional descriptors, topological descriptors, walk and path count descriptor, connectivity indices, information indices, 2D autocorrelation, edge adjacency, burden eigenvalues, topological charge indices, functional groups, molecular properties and eigenvalues based indices. Initially, the descriptors with zero or unassigned values were excluded and then pair-wise correlation test to remove highly correlated descriptors at a cut-off value of 0.50 was executed. This procedure resulted in 14 descriptors for 2D QSAR modeling.

### QSAR Model Construction

QSAR methodology quantitatively correlates the structural molecular properties (descriptors) with functions (biological activities) for a set of compounds by means of linear or non-linear statistical methods. In the present study, we exploited both linear (MLR) and non-linear (SVM) statistical methods for flourishing the robust QSAR models [27,28]. Retrospectively, for QSAR modeling, both linear and non-linear models have been extensively used [29-36]. MLR tries to model the relationship between two or more independent descriptors and dependent variable such as *K*_i _by fitting a linear regression equation to the observed data with corresponding parameters (constants) and an error term. On the other hand, SVM based on statistical and optimization theory, handles complex structural features. In the present study, SVM_light http://www.cs.cornell.edu/People/tj/svm_light/, which is an implementation of SVM, was used for QSAR modeling.

### Evaluation of QSAR models

To assess the predictive performance of QSAR models, different cross-validation procedures were adopted. First, in leave-one-out strategy (LOOCV), one molecule was removed from the dataset as a test compound and the remaining 22 molecules were used to build the model. This process was repeated 23 times with each inhibitor as a test molecule. Once a regression model was constructed, goodness about the fit and statistical significance was assessed using the statistical parameters outlined below(1)

where, *x*_*i *_and *y*_*i *_represents the actual and predicted *K*_i _values for the *i*^th ^compound. *N *is the total number of compounds,  represents the averaged value of the actual *K*_i _for the entire dataset.

Here, it was equally important to use an independent test set to check the real predictive accuracy of trained QSAR models. However, 23 compounds were not expected to be sufficient for independent testing using existing QSAR models. Therefore, an alternative strategy, external cross-validation, was adopted, where different number of inhibitors i.e. 2, 4, and 6 were randomly selected as independent test sets. The models were then trained on the remaining inhibitors i.e. 21, 19, and 17 using LOOCV procedure followed by independent testing on the corresponding test sets. This cycle of randomly separating test and training sets was repeated. Here, to determine the predictive accuracy of models on the test set, predictive *r*^2 ^value was used(4)

where, SD is the sum of the squared deviations between the activities of the test set and mean activities of the training molecules.

Then, Y-randomization test was performed in order to appraise high training and testing correlation values observed during QSAR modeling, were not occurred incidentally. Here, the shuffled activity dataset was derived by randomly shuffling the dependent variables *K*_i _and keeping the descriptors original, afterward using this randomly shuffled dataset to develop new QSAR models. The process of shuffling was carried out many times with subsequent generation of corresponding models nevertheless, with an assumption that the resulting models should give low performance, which would obviously imply the rigorous robustness of the original models.

### Input variables selection

The selection of best descriptors that establish the relationship between chemical structure and an inhibitory property is crucial for the success of QSAR modeling. In the present study, we adopted F-stepping remove-one approach for variable selection. Accordingly, each input variable was removed one-by-one from the set of *n *variables followed by QSAR modeling using the remaining *n*-1 variables. However, if the correlation value was increased, the particular variable was permanently removed from the analysis. These cycles were repeated until no further improvement in the correlation values was observed and stopped if *n*-1 removal resulted in reduction of correlation values.

## List of abbreviations used

The abbreviations used are: QSAR: Quantitative Structural Activity Relationship; DAP: Diaminopimelic Acid; CADD: Computed Aided Drug Designing; LYS161: Lysine-161; SVM: Support Vector Machine; LOOCV: Leave-One-Out Cross-Validation; MAE: Mean Absolute Error; MLR: Multiple Linear Regression.

## Competing interests

The authors declare that they have no competing interests.

## Authors' contributions

AG, GPSR and RT conceived and designed the experiments. AG performed the experiments, wrote perl scripts, developed server. GPSR and AG analyzed the data. AG wrote the manuscript. AG and GPSR carried out revision of the manuscript. This manuscript has been seen and approved by all authors.

## Availability and requirements

Project name: A webserver for predicting DHDPS inhibitors

Project home page: http://crdd.osdd.net/raghava/kidoq

Operating system(s): Platform independent;

Programming language: PERL, CGI-PERL;

License: None;

Any restrictions to use by non-academics: No restrictions

## Supplementary Material

Additional file 1 **Textbox S1: Selection of descriptors based on the chemical structures and activities**. Additional file shows the descriptor selection on the basis of similarity in chemical structure of inhibitors and their activity. Click here for file
