# Supplementary material for: KiDoQ: using docking based energy scores to develop ligand based model for predicting antibacterials
Source: BMC Bioinformatics. 2010 Mar 11;11:125. doi: 10.1186/1471-2105-11-125 (PMC2841597; doi:10.1186/1471-2105-11-125)
Supplement: Additional file 1 — Textbox S1: Selection of descriptors based on the chemical structures and activities. Additional file shows the descriptor selection on the basis of similarity in chemical structure of inhibitors and their activity. [file 1471-2105-11-125-S1.DOC]

**Textbox S1: Selection of descriptors based on the chemical structures and activities.**

The present model was developed on a diverse dataset consisted of inhibitors, which were mainly the analogues of substrate pyruvate, intermediate and product DHDP. Interestingly, removing the outliers, the remaining inhibitors belonging to above-mentioned three different classes were demonstrating different binding patterns, which could be noticed in terms of binding free energy values. For instance, pyruvate analogues (such as Inh-1, Inh-3, Inh-4, Inh-5, Inh-6, and Inh-15) particularly Inh-4 and Inh-6 the electrostatic interaction was observed to be very weak or absent, hence, the affinity of these inhibitors was presumed to be highly dependent on hydrogen bonding and other non-bonded interactions such as VDW and hydrophobic. For Inh-6 the presence of carboxyl and ketone groups were able to form utmost six hydrogen bonds between active side residues such as THR45, LYS161, and TYR133. Here, an additional ketone group at C-4 position facilitated the formation of an additional hydrogen bond with TYR133-OH merely at the distance of 1.835Å; hence, making the binding more stronger and provided lower *K*_i_ value of 0.005mM in comparison to other pyruvate analogues. Similarly, presence of carboxyl and ketone group (at C-2 position) in Inh-4 were found to be invloved in 5 hydrogen bondings with THR45, LYS161, and THR44 residues, wheras Br was involved only in VDW interactions. Thus, hydrogen bonding appeared to be a dominant interaction and provided E_FreeBind_ values of -7.17 and -7.15 (kcal/mol) respectively for these two inhibitors. Besides, other non-bonded interactions such as VDW and hydrophobic were imperative in stabilizing and holding the bound conformations.

The inhibitory activity values for Inh-1 and Inh-3 was found to be comparable, and the same trend was observed in their predicted E_FreeBind_. Both inhibitors differ from the pyruvate by the presence of methyl and ethyl groups at C-3 position respectively. Despite of a little increment in size binding interactions and predicted E_FreeBind_ values were found to be same. Hence, increasing the size of an analogue by adding aliphatic chain at C-3 position observed to be irrelevant to enhance the affinity for DHDPS. The dominant interactions were observed to be hydrophobic and VDW besides four hydrogen bonds with THR45, LYS161, and THR44 residues. Therefore, for the pyruvate analogues, we suggest that addition of charged polar groups rather than hydrophobic and aliphatic chains may be beneficial in providing stronger and favourable interactions.

Further, Inh-15 (an analogue smaller than pyruvate) and Inh-5 were found to be interacted with receptor by forming 5 and 6 hydrogen bonds at active site residues of DHDPS respectively. But the absence of hydrophobic interactions and few VDW interactions resulted in the weak binding, hence provided low E_FreeBind_. Further, docking of Inh-18 was characterized by hydrogen bond formation with THR45, LYS161 and ASN248; strong electrostatic interaction with LYS161 residue at a distance of 2.8Å along with high hydrophobic interactions which eventually provided high negative E_FreeBind_ value. However, in Inh-9 an analogue of Inh-18, the presence of charged carboxyl group at C-4 position enhanced the binding by setting strong hydrogen bonding with ARG138, THR45, LYS161 and ASN248 residues; electrostatic interaction with LYS161 residue at a distance of 2.8Å, VDW and hydrophobic interactions, which eventually yielded high negative E_FreeBind_ in comparison to Inh-18. Herein, the predicted free energy binding E_FreeBind_ value was observed to be in compliance with *K*_i_ value for both inhibitors.

Furthermore, two inhibitors such as Inh-2 and Inh-11 differ only by the presence of ketone group at C-6 position in later; however, addition of this group could not produce profound effect on the inhibition as both inhibited the enzyme with same *K*_i_ values of 0.17 and 0.16mM respectively. Hence, as expected approximately the same E_FreeBind_ values of -7.52 and -7.50 (kcal/mol) was also obtained while docking of these two inhibitors respectively. Interestingly, docking of Inh-11 was distinguished by strong electrostatic interactions between ARG138 side chain and ligand’s ketone group at C-6 position yielding high negative E_Elec_ value of -0.99; hydrogen bond formation with THR45, THR44, LYS161 and ARG138 along with VDW interactions. On the other hand no electrostatic interactions were observed in Inh-2, however, absence of electrostatic interactions were overcome by strong hydrogen bonding with THR45, THR44, LYS161, TYR133 and ARG138 residues along with strong hydrophobic interactions, which accomplished the docking of Inh-2 as strong as Inh-11.

Besides, other inhibitors included analogues of DHDP and intermediates which were aromatic and few pyridines, yielding E_FreeBind_ value in the range of -7.5 to -8.68 (kcal/mol). Here, docking was found to be very strong with high negative E_FreeBind_ values for Inh-19, Inh-13, and Inh-14, which were designed to mimic DHDP. Unfortunately, among these three inhibitors, binding of Inh-14 a pyridine molecule which consisted of two carboxyl groups at C-2 and C-6 positions was found to be overestimated in terms of high negative E_FreeBind_ value but experimentally inhibited the enzyme with higher *K*_i_ value of 11mM. The thorough exploration of this mismatch revealed that the bound conformation is exclusively characterized by *π*-cationic interactions depicting E_Elec_ value of -0.93 (kcal/mol). Inspite of strong hydrogen bonding and electrostatic interactions, the internal energy value of the receptor was observed to be low (E_IntR_ -2.01 kcal/mol) in comparison with other inhibitors. Presumably, the binding of inhibtor due to strong *π-cationic* electrostatic interaction might have destabilized the receptor, which resulted in the low E_IntR_. Thus, inhibitor has the capability to form strong binding with receptor as docking yielded a high negative value of E_FreeBind_, however, at the cost of destablizing receptor structure, which can be assured from the low negative E_IntR_ value, hence all-together provided overestimated negative value of E_FreeBind_ not in correlation with *K*_i_ value. Therefore, a proof that sometimes a higher negative value of E_FreeBind_ might occur as a matter of chance, but E_IntR_ of the receptor that determine the stability receptor is another crucial criteria which should be also verified to filter the false positive hits. For these reasons, it can be presumed that in pyridine analogues electrostatic interactions, particularly, *π*-cationic interactions destabilize the bound conformations and provided the overestimated value of E_FreeBind_. Comparatively, other analogues Inh-19 and Inh-13 were predicted with high negative E_IntR_ of the receptor such as -2.82 and -2.71 (kcal/mol) respectively hence, providing highly stable bound conformations. Both compounds were similar to Inh-14; however, former observed to be characterized by the presence of additional oxide group at N, whereas in the later, CN groups replaced both carboxyl groups. Importantly, electrostatic interactions was not observed in both of these inhibitors, nevertheless, strong hydrogen bonding, VDW and hydrophobic interactions made the binding of Inh-19 stronger in comparison with Inh-13. Interestingly E_FreeBind_ values were found to be in accordance with *K*_i_ value for both inhibitors. The main reason for the strong binding of highly charged Inh-19 in comparison to Inh-13 was due to the formation of additional hydrogen bonding in the former as a result of two carboxyl groups present at C-2, C-6 positions and the oxide group on N atom which caused an inherent charge separartions with a partial positive charge on N atom and partial negative charge on O atom. Whereas, in the case of Inh-13, an occurrence of CN groups in the place of carboxyl group and removal of N-oxide group reduced the interactions sites with receptor’s residues, hence resulted in lesser stronger binding in comparison to Inh-19. Similarly, other aromatic inhibitors such as Inh-10, Inh-12, and Inh-17 showed the same binding pattern as Inh-14.

**Table S1: Different binding poses with predicted E_FreeBind_ and RMSD values for substrate pyruvate**

| ***Rank*** | ***E_FreeBind_ (kcal/mol)*** | ***RMSD(Å)*** |
| --- | --- | --- |
|  | **-6.92** | **0.35** |
|  | -6.73 | 0.42 |
|  | -6.72 | 0.48 |
|  | -6.7 | 0.46 |
|  | -6.67 | 0.27 |
|  | -6.52 | 0.54 |
|  | -6.46 | 0.96 |
|  | -6.45 | 0.92 |
|  | -6.39 | 0.55 |
|  | -6.27 | 1.17 |
